# Supplementary material for: Spatial dependence in the rank-size distribution of cities – weak but not negligible
Source: PLoS One. 2021 Feb 9;16(2):e0246796. doi: 10.1371/journal.pone.0246796 (PMC7872244; doi:10.1371/journal.pone.0246796)
Supplement: Attachment — Submitted filename: PLOS D-20-16834 (1).pdf [file pone.0246796.s002.pdf]

Referee's Report on ms. #20-16834
"Spatial dependence in the rank-size distribution of cities"
by R. Bergs

This paper examines, mainly empirically, the role of spatial dependence in Zipf's and related laws. The theory consists of a gravity law for city size and a Zipf regression, where distance and rank of neighboring cities are included in the Zipf-style regression. Simulations reveal that a statistical model with a Pareto top and lognormal bottom eliminates spatial dependence. Real world data reveals not only this relationship but also residual spatial dependence.

There is much to say about this work. First, let's begin with some context. There is really no economic model here; the model is purely statistical. The gravity model is not derived in this context from primitives, and is rather hokey. This is, in a way, a step backward. The pre-Gabaix and post-Gabaix periods are distinguished by the presence of economic models in the post-Gabaix work. The problem with these models is often that they are designed to generate Zipf-like laws, and nothing else.

Second, this is not the first paper to notice the transition between Pareto at the top of the distribution and lognormal at the bottom (lines 55-60): Ioannides and Skouras, *JUE*, 2013. Not even cited.

Third, there is a theory for why distance should matter in these Zipf-style regressions, called Central Place Theory, which is almost as famous as Zipf's law. It is the long-time pursuit of Wen-Tai Hsu. See, for a recent example, Mori, Smith and Hsu, *PNAS*, 2020, 117 (12) 6469-6475.

Fourth, given the spatial dependence in rank R (the dependent variable), I wonder if there is also spatial dependence in population S, the independent variable.

Fifth, the structure of spatial dependence in the regression should really be derived from the underlying economics.
