# Supplementary material for: Spatial dependence in the rank-size distribution of cities – weak but not negligible
Source: PLoS One. 2021 Feb 9;16(2):e0246796. doi: 10.1371/journal.pone.0246796 (PMC7872244; doi:10.1371/journal.pone.0246796)
Supplement: Attachment — Submitted filename: PONE-D-20-16834 (2).pdf [file pone.0246796.s004.pdf]

PLOS ONE

Spatial dependence in the rank-size distribution of cities

--Manuscript Draft--

**Manuscript Number:**	PONE-D-20-16834
**Article Type:**	Research Article
**Full Title:**	Spatial dependence in the rank-size distribution of cities
**Short Title:**	Spatial dependence
**Corresponding Author:**	Rolf Bergs, Ph.D.<br>PRAC<br>Bad Soden, Hessen GERMANY
**Keywords:**	Spatial dependence; Zipf's law; spatial econometrics
**Abstract:**	Power law distributions characterise several natural and social phenomena. Zipf's law for cities is one of those. The study views the question of whether that global regularity is independent of different spatial distributions of cities. For that purpose, a typical Zipfian rank-size distribution of cities is generated with random numbers. This distribution is then cast into different settings of spatial coordinates. For the estimation, the variables rank and size are supplemented by considerations of spatial dependence within a standard spatial econometric approach. Results suggest that distance matters. This finding is further corroborated by three country analyses.
**Order of Authors:**	Rolf Bergs, Ph.D.
**Opposed Reviewers:**	
**Additional Information:**	
**Question**	**Response**
**Financial Disclosure**<br><br>Enter a financial disclosure statement that describes the sources of funding for the work included in this submission. Review the submission guidelines for detailed requirements. View published research articles from *PLOS ONE* for specific examples.<br><br>This statement is required for submission and **will appear in the published article** if the submission is accepted. Please make sure it is accurate.	Funding was provided by the European Commission via its Horizon2020 research funding (https://cordis.europa.eu/project/id/727988/de). The funders had no role in study design, data collection and analysis, decision to publish, or preparation of the manuscript.

Powered by Editorial Manager® and ProduXion Manager® from Aries Systems Corporation

**Unfunded studies**<br>Enter: *The author(s) received no specific funding for this work.*<br><br>**Funded studies**<br>Enter a statement with the following details:<br>• Initials of the authors who received each award<br>• Grant numbers awarded to each author<br>• The full name of each funder<br>• URL of each funder website<br>• Did the sponsors or funders play any role in the study design, data collection and analysis, decision to publish, or preparation of the manuscript?<br>• **NO** - Include this sentence at the end of your statement: *The funders had no role in study design, data collection and analysis, decision to publish, or preparation of the manuscript.*<br>• **YES** - Specify the role(s) played.<br><br>* typeset	
**Competing Interests**<br><br>Use the instructions below to enter a competing interest statement for this submission. On behalf of all authors, disclose any competing interests that could be perceived to bias this work—acknowledging all financial support and any other relevant financial or non-financial competing interests.<br><br>This statement **will appear in the published article** if the submission is accepted. Please make sure it is accurate. View published research articles from *PLOS ONE* for specific examples.	The author has declared that no competing interests exist.

**NO authors have competing interests**<br><br>Enter: *The authors have declared that no competing interests exist.*<br><br>**Authors with competing interests**<br><br>Enter competing interest details beginning with this statement:<br><br>*I have read the journal's policy and the authors of this manuscript have the following competing interests: [insert competing interests here]*<br><br>* typeset	
**Ethics Statement**<br><br>Enter an ethics statement for this submission. This statement is required if the study involved:<br><br>• Human participants<br>• Human specimens or tissue<br>• Vertebrate animals or cephalopods<br>• Vertebrate embryos or tissues<br>• Field research<br><br>Write "N/A" if the submission does not require an ethics statement.<br><br>General guidance is provided below. Consult the submission guidelines for detailed instructions. **Make sure that all information entered here is included in the Methods section of the manuscript.**	N/A

Powered by Editorial Manager® and ProduXion Manager® from Aries Systems Corporation

**Format for specific study types**<br><br>**Human Subject Research (involving human participants and/or tissue)**<br>• Give the name of the institutional review board or ethics committee that approved the study<br>• Include the approval number and/or a statement indicating approval of this research<br>• Indicate the form of consent obtained (written/oral) or the reason that consent was not obtained (e.g. the data were analyzed anonymously)<br><br>**Animal Research (involving vertebrate animals, embryos or tissues)**<br>• Provide the name of the Institutional Animal Care and Use Committee (IACUC) or other relevant ethics board that reviewed the study protocol, and indicate whether they approved this research or granted a formal waiver of ethical approval<br>• Include an approval number if one was obtained<br>• If the study involved *non-human primates*, add *additional details* about animal welfare and steps taken to ameliorate suffering<br>• If anesthesia, euthanasia, or any kind of animal sacrifice is part of the study, include briefly which substances and/or methods were applied<br><br>**Field Research**<br><br>Include the following details if this study involves the collection of plant, animal, or other materials from a natural setting:<br>• Field permit number<br>• Name of the institution or relevant body that granted permission	
**Data Availability**<br><br>Authors are required to make all data underlying the findings described fully available, without restriction, and from the time of publication. PLOS allows rare exceptions to address legal and ethical concerns. See the PLOS Data Policy and FAQ for detailed information.	Yes - all data are fully available without restriction

A Data Availability Statement describing where the data can be found is required at submission. Your answers to this question constitute the Data Availability Statement and **will be published in the article**, if accepted.<br><br>**Important:** Stating 'data available on request from the author' is not sufficient. If your data are only available upon request, select 'No' for the first question and explain your exceptional situation in the text box.<br><br>Do the authors confirm that all data underlying the findings described in their manuscript are fully available without restriction?	
**Describe where the data may be found in full sentences. If you are copying our sample text, replace any instances of XXX with the appropriate details.**<br><br>• If the data are **held or will be held in a public repository**, include URLs, accession numbers or DOIs. If this information will only be available after acceptance, indicate this by ticking the box below. For example: *All XXX files are available from the XXX database (accession number(s) XXX, XXX.*).<br>• If the data are all contained **within the manuscript and/or Supporting Information files**, enter the following: *All relevant data are within the manuscript and its Supporting Information files.*<br>• If neither of these applies but you are able to provide **details of access elsewhere**, with or without limitations, please do so. For example:<br><br>*Data cannot be shared publicly because of [XXX]. Data are available from the XXX Institutional Data Access / Ethics Committee (contact via XXX) for researchers who meet the criteria for access to confidential data.*<br><br>*The data underlying the results presented in the study are available from (include the name of the third party*	All relevant data are within the manuscript and its Supporting Information files.

and contact information or URL).<br>• This text is appropriate if the data are owned by a third party and authors do not have permission to share the data.<br><br>* typeset	
Additional data availability information:	

Powered by Editorial Manager® and ProduXion Manager® from Aries Systems Corporation

Dr Rolf Bergs
PRAC Bergs & Issa Partnership Co.
Im Hopfengarten 19b
65812 Bad Soden
Germany

3 June 2020

Dr Joseph Donlan
Associate Editor, Physical Sciences, PLOS ONE
Carlyle House, Carlyle Road, Cambridge CB4 3DN
United Kingdom

Dear Dr Donlan,

further to our communication from mid-May regarding the Call for Papers on the topic of „Cities as Complex Systems“ I herewith submit my paper on „Spatial dependence in the rank-size distribution of cities“. The manuscript is original and was not submitted elsewhere. As you know, the paper had been uploaded as a preprint on arXiv on 7 May, 2020.

The research for that was carried out in the context of a current EU research project (Horizon2020 research grant 727988).

I would be pleased if the paper is deemed of sufficient quality and relevance.

Yours sincerely,

Rolf Bergs

Click here to download Manuscript Paper_Spatial dependence.docx

Spatial dependence in the rank-size distribution of cities

Rolf Bergs

PRAC, Im Hopfengarten 19b, 65812 Bad Soden, Germany. E-Mail: RolfB@prac.de

Abstract

Power law distributions characterise several natural and social phenomena. Zipf's law for cities is one of those. The study views the question of whether that global regularity is independent of different spatial distributions of cities. For that purpose, a typical Zipfian rank-size distribution of cities is generated with random numbers. This distribution is then cast into different settings of spatial coordinates. For the estimation, the variables rank and size are supplemented by considerations of spatial dependence within a standard spatial econometric approach. Results suggest that distance matters. This finding is further corroborated by three country analyses.

JEL-Classification: O18; R12

1. Introduction

Zipf's law of the rank-size distribution of cities is regarded as an enthrallment of rare social physics. Krugman has described this phenomenon even as an embarassment for economic theory [1: 42-46]. At first glance, the relationship between size and rank suggests a tautological relationship because size directly determines rank and *vice versa*. The independent variable is thus perhaps no true predictor but could just reflect a simple universal statistical phenomenon. The power law exponent typically close to -1 and a determination coefficient (R^2) close to one are an indication of that. Therefore some have emphatically questioned the relevance for economic analysis, notably Gan et al. [2]. Yet, such a striking ubiquitous regularity has motivated the exploration of hidden explanatory factors behind. Various authors, such as Gabaix, Fujita et al., Brakman et al. and Reggiani and Nijkamp have done this [3, 4, 5, 6]. In contrast to the *Zipf* distribution of frequency of words in languages [7], the rank-size distribution of cities appears slightly more varied between countries as shown by Rosen and Resnick [8] and less stable over time as found by Brakman et al. [9] but there is a secular convergence that is explained by *Gibrat's* law and its resulting steady state [3]. The fact that this happens in all countries, regardless their economic structures and histories,

still lacks a sufficient explanation. When comparing such power law distributions for different types of data one criterion could perhaps add minor insight: A spatial versus non-spatial context. Contiguity or distance between cities of different size may affect *Zipf's* law for cities; in contrast, space can never predict the rank-distribution of words.

To have a closer look at that context, I demonstrate a typical rank-size distribution of cities for a varied distribution of spatial coordinates. The objective is to see how much influence spatial distance could have on the ranks of cities and thus the shape of the distribution. Different levels of concentration and dispersion of urban space among countries suggest such an exploration. Especially in countries with a geographical concentration of bigger cities there is reason to assume that these cities have evolved due to certain spatial advantages (e.g. raw materials, climate, accessibility or certain random determinants). Those city clusters are often characterised by specific industries of national importance. Whether and how dispersion and concentration forces determine the rank-size distribution of cities has been an object of research in urban economics.

Surprisingly, there has been little research shedding light on the spatial dimension of *Zipf's* law. Lalanne views the dichotomic urban structure of Canada [10]. She rejects the *Zipf* law and its underlying scale invariance and shows that the Canadian urban structure has evolved in a deterministic process based on urban size (inhabitants within administratively defined boundaries), previous growth and the spatial setting. Coefficients for the years 1971 to 2001 vary between -0.77 and -0.81. The spatial component is not part of the *Pareto* regression; instead growth of cities is regressed on size and previous growth in standard spatial regression models (SEM and SAR). Rybski et al. simulate urban evolution under the assumption that urban growth takes place close to other urban settlements [11]. They confirm *Zipf's* law and scale invariance of the clusters generated, except the primate one. Le Gallo and Chasco explore *Zipf's* law for Spain by applying a SUR model which they cast into spatial autoregressive and error specifications. *Zipf's* law does not hold between 1900 and 2001. While the simple OLS estimate varies between -0.54 and -0.66, the extended spatial models deviate even further from *Zipf's* law, thus revealing spatial impacts [12]. Cheng and Zhuang [13], who look at urban evolution in central China under consideration of *Zipf's* law, show that the estimation of the *Pareto* coefficient has displayed an undulatory pattern between 1985 and 2009. They cannot confirm *Zipf's* law at any point of time. The OLS estimates are then augmented by the

use of spatial autoregressive or spatial error specifications. Like in the study of Le Gallo and Chasco [12], spatial dependence increases the deviation from *Zipf's* law. It is, however, to be stressed that in the three studies on Canada, Spain and China size of cities is not defined by functionality but the number of inhabitants within administratively defined boundaries of all cities, i.e. including the lower tail of the distribution. This is a reason why *Zipf's* law often does not hold [5: 301-306].

In the study at hand I intend to show (i) whether and how spatial effects vary by concentration and dispersion and (ii) how they behave differently along the entire distribution of cities and specifically the upper *Pareto* tail. Evidence suggests that the rank-size distribution is not homogeneously following a *Pareto* shape, but rather a combination of an upper *Pareto* and a lower lognormal section. In essence, the paper is not a real world study but rather draws on a theory-led artificial simulation of *Zipf's* law. For this purpose, I random-generate a typical database of city rank-size distribution with both, an upper *Pareto* as well as a lower lognormally distributed tail. Here I assume the regularity, several authors have demonstrated empirically (a known controversy is: Malevergne et al. 2011 [14] versus Eeckhout 2004 [15]. Different patterns of spatial distribution of cities are then explored. Finally, the simulation exercise is complemented by three country data sets of natural cities which had been detected by statistical segmentation of night satellite images. Such natural cities represent the true functional urban space much more precisely than cities proper. The original purpose of term „Natural Cities“, formulated from an architectural viewpoint by Alexander [16], was to distinguish between planned cities and urban space evolved over time. In the present debate, novel databases such as nocturnal satellite imagery or social media data are used to view spatial heterogeneity and dependence in such natural urban systems [17, 18, 19].

2. Methodological approach

The central assumption is as follows: For the typical upper *Pareto (3,1)* tail the expected exponent α is approximately 1 (±0.1) as empirically confirmed ubiquitously. The relationship in terms of a cumulative distribution function is given by:

$$R = CS^{-\alpha}$$

or in its log-linear form:

$$\ln(R) = \ln(C) - \alpha \ln(S)$$

where R is rank, S means size and C is a constant. OLS or maximum likelihood are possible estimators of α. For the combined *Pareto* and lognormal tails the exponent does not fit *Zipf's* law as usual. Empirically only the upper tail of bigger cities follows a Zipfian distribution. It is however expected that, in accordance with the gravity model, interaction between two places (cities) depends on their mass *M* and their distance *D* so that interaction decreases with distance: $I_{j,k} = GM_jM_kD_{j,k}^{-\gamma}$. G is a proportionality coefficient and γ an exponent showing the distance decay of interaction. This gravity relationship, known from international trade, also represents *Tobler's* law in its sense that "everything is related to everything else, but near things are more related than distant things..." [20]. Gravity is however not incorporated into *Zipf's* law, so that the *Pareto* exponent α≈1 in one country may remain stable under consideration of spatial interaction while, in another one, the incorporation of such interaction could perhaps lead to major deviations of α. This assumption suggests to further test spatial dependence in *Zipf's* law for cities by the following alternative spatial econometric procedures (spatial autoregressive versus spatial error model) where D_{jk} is included through a spatial weight matrix:

$$\ln(R) = \rho\omega \ln(R) + \ln(C) - \alpha \ln(S) + \varepsilon \qquad (\mathbf{SAR})$$

or

$$\begin{cases} \ln(R) = \ln(C) - \alpha \ln(S) + \nu \qquad (\mathbf{SEM}) \\ \nu = \lambda\omega\nu + \varepsilon \end{cases}$$

where ω is a N x N row-standardised weight matrix (inverse distance) to capture a potential distance effect and *C* is a constant while ρ (spatial spillover) and λ (spatial dependence in the residuals) in addition to α (direct effects) are the coefficients estimated. The error term ν in the SEM case consists of spatial error and the residual $\varepsilon \backsim N(0, \sigma^2)$.

The right choice between both models can be determined by different tests, such as the z-score of *Moran's I* of the residuals and (Robust) *Lagrange* multiplier (LM) tests. The different estimation types in Tables 1 and 2 correspond to the respective choice.

The simulated „countries“ describe a distribution with in each case 109 observations for cities, the upper 50 being *Pareto* (3,1) distributed and the lower 59 with a lognormal shape. Both distributions are random-generated and then matched into one data set. This hybrid rank-size distribution is then combined with different distributions of coordinates X and Y, the first one being randomly generated to fit a normal distribution. Based on this configuration a spatial weight matrix is derived. With a normal distribution of both X and Y coordinates big and small cities are spread evenly.

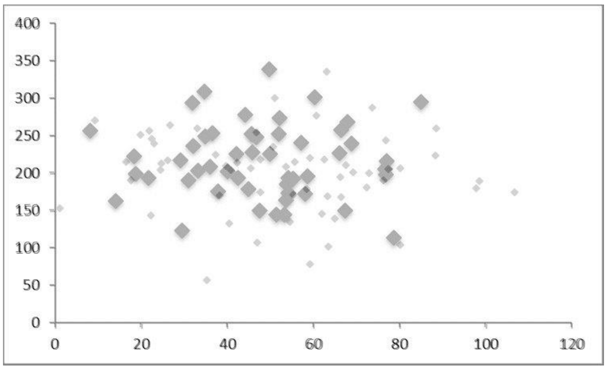

Fig. 1: Normal spatial distribution of cities

In a second variation with the same rank distribution the normally distributed coordinates of X and Y are both ranked as well, so that all cities are geographically positioned on a diagonal line, ordered along rank, the biggest city in the outer North-East, the smallest one in the outer South-West (like a one-dimensional *von-Thunen* assembly).

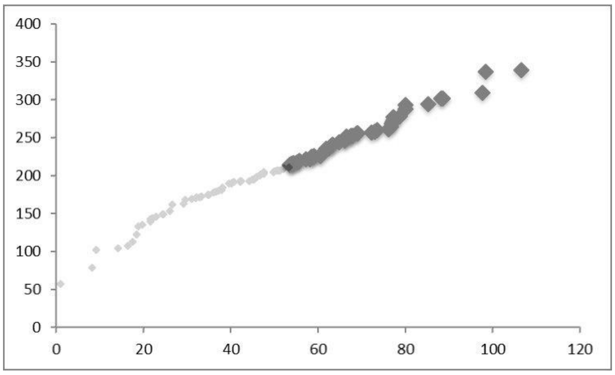

Fig. 2: Rank-size distribution of cities with ranked coordinates

It is expected that in this case both, rank as well as size, exhibit spatial autocorrelation of city size even though such a setting is hardly encountered in the real world. In a third data set both, coordinates of the upper tail (*Pareto*) as well as the lower tail cities (lognormal) are normally

distributed, however geographically separated and within a larger spatial frame, where coordinates of the upper tail are changed to 2X and 2Y. Here an indication of spatial dependence is likewise expected, but rather for the entire distribution.

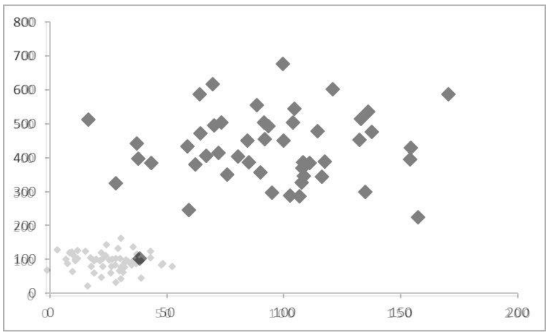

Fig. 3: Dichotomous spatial distribution of big and small cities

In a fourth variant there is a center-periphery pattern, however X coordinates of the bigger cities are again ranked so that the assembly follows a geographical distribution of biggest cities in the East, while the small cities remain normally distributed over space.

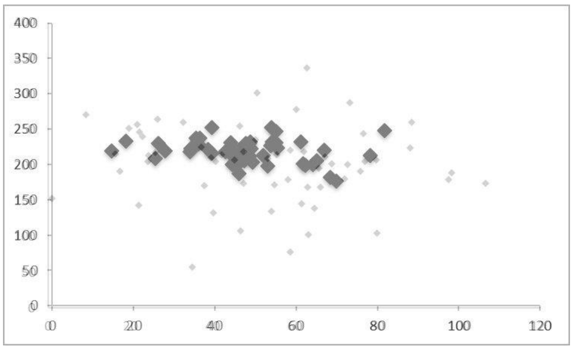

Fig.4: Non-normally distributed big cities and normally distributed small cities

Variants II, III and IV represent excessive non-normal formings and are hardly encountered in the real world; the only purpose of these extreme settings is to show the possible potential of spatial impact depending on the distribution of coordinates. With other words, identical coefficients confirming *Zipf's* law may have a different meaning for different countries.

All simulated cases are then compared with respect to the stability of α and the significance of the spatial parameters ρ or λ respectively. I hypothesise that with a normally distributed arrangement of coordinates the spatial weight parameters are insignificant and meaningless, representing a *Zipf* distribution of the upper tail similar to that of words in a language. However, when modifying the coordinates and the spatial distribution of cities by building clusters of cities with a different level

of size we may expect some stronger and significant spatial impact. The interesting question is then how stable the original *Zipf* distribution of the upper tail remains.

Finally I examine the spatial influence on *Zipf's* law with Dutch, Slovene and Austrian natural cities extracted from night satellite images assuming that those better represent the true urban space as compared to the number of inhabitants within administratively defined city boundaries. The background and the image segmentation methodology are futher explained in Bergs [19] and Bergs and Issa [21]. The source of data is the National Oceanic and Atmospheric Administration (NOAA) [22].

3. Results

Table 1 shows the full rank-size distribution, while Table 2 displays the estimates only for the upper (*Pareto*) tail. First I take a look at the different results of the simulation exercise. Regarding case I, *Zipf's* law is only confirmed for the curtailed distribution: $\alpha \approx 1$ (Table 2). The lognormal extension with the smaller cities reduces the estimate of α to a large extent. As expected, the spatial coefficients λ of the full distribution and ρ of the *Pareto* tail are not significant. The normal distribution of coordinates of big and small cities leads to zero spatial dependence (*Moran's* I of the variable *Area* is close to zero). With or without the spatial extension of the model the α coefficient remains the same. *Zipf's* law is well confirmed for the *Pareto* tail.

Table 1: Spatial extension of Zipf's law (full rank-size distribution)

Endogenous variable: ln(Rank)	I	II	III	IV	The Netherlands 2011 (DMSP)	Slovenia 2017 (VIIRS)	Austria 2017 (VIIRS)
ln(Area)	-0.403	-0.322	-0.321	-0.432	-0.424	-0.739	-0.833
(Standard error)	(0.024)***	(0.028)***	(0.033)***	(0.026)***	(0.026)***	(0.124)***	(0.007)***
Constant	3.871	1.057	2.355	3.620	5.300	6.240	7.310
(Standard error)	(0.037)***	(2.998)***	(0.435)***	0.483)***	(0.787)***	(0.742)***	0.120)***
λ	-0.321	0.969		0.882	-	-	0.031
(Standard error)	(0.650)	(0.030)***		(0.117)***	-	-	(0.202)
ρ	-	-	0.392	-	-0.159	-0.110	-
(Standard error)	-	-	(0.112)***	-	(0.228)	(0.165)	-
Log Likelihood	-78.581	-44.774	-72.992	-72.750	-37.566	2.579	28.147
Wald-Test λ or ρ = 0 (χ^2)	0.244	1016.678	12.293	57.052	0.484	0.441	0.023
(p)	(0.622)	(0.000)	(0.000)	(0.000)	(0.487)	(0.507)	(0.879)
z-score of Moran's I (resid.)	0.141	10.081	-	7.561	-	-	0.407
(p)	(0.888)	(0.000)	-	(0.000)	-	-	(0.684)
Lagrange Multiplier (LM)	0.163	80.748	14.883	19.539	0.310	0.436	0.027
(p)	(0.686)	(0.000)	(0.000)	(0.000)	(0.578)	(0.509)	(0.870)
Robust LM	0.028	15.462	11.544	39.497	0.260	0.671	0.042
(p)	(0.867)	(0.000)	(0.001)	(0.000)	(0.610)	(0.413)	(0.838)
Moran's I (Area)	0,014	0,473	0.429	0.123	0.053	-0.016	0.010
(p)	(0,039)	(0,000)	(0.000)	(0.000)	(0.043)	(0.095)	(0.038)
Obs.	109	109	109	109	71	243	644
OLS							
ln(Area)	-0.403	-0.403	-0.403	-0.403	-0.421	-0.741	-0.833
(Standard error)	(0.025)***	(0.025)***	(0.025)***	(0.025)***	(0.026)***	(0.123)***	(0.008)***
Constant	3.870	3.870	3.870	3.870	4.757	5.748	7.310
(Standard error)	(0.049)***	(0.049)***	(0.049)***	(0.049)***	(0.102)***	(0.258)***	(0.020)***
Adj. R^2	0.709	0.709	0.709	0.709	0.789	0.937	0.944

Table 2: Spatial extension of Zipf's law (rank-size distribution restricted on the upper tail)

	I	II	III	IV	The Netherlands 2011 (DMSP)	Slovenia 2017 (VIIRS)	Austria 2017 (VIIRS)
Endogenous variable: ln(Rank)							
ln(Area)	-0.995	-0.862	-0.995	-0.986	-0.905	-0.860	-0.966
(Standard error)	(0.017)***	(0.029)***	(0.018)***	(0.019)***	(0.070)***	(0.171)***	(0.022)***
Constant	5.086	3.846	5.117	4.937	7.202	6.540	7.482
(Standard error)	(0.430)***	(0.208)***	(0.423)***	(0.095)***	(0.390)***	(0.253)***	(0.104)***
λ	-	-	-	0.813	-0.679		-0.731
(Standard error)	-	-	-	(0.180)***	(0.919)		(0.345)*
ρ	-0.056	0.262	-0.067	-	-	-0.550	-
(Standard error)	(0.145)	(0.050)***	(0.144)	-	-	(0.240)*	-
Log Likelihood	39.618	50.343	39.652	42.830	-2.167	8.389	28.731
Wald-Test λ or ρ = 0 (χ^2)	0.149	27.289	0.218	20.355	0.547	5.255	4.478
(p)	(0.700)	(0.000)	(0.640)	(0.000)	(0.460)	(0.022)	(0.034)
z-score of Moran's I (resid.)	-	-	-	5.072	0.148	-	-1.531
(p)	-	-	-	(0.000)	(0.882)	-	(1.874)
Lagrange Multiplier (LM)	0.146	23.817	0.213	8.054	0.287	3.465	2.764
(p)	(0.703)	(0.000)	(0.645)	(0.005)	(0.592)	(0.063)	(0.096)
Robust LM	0.081	14.067	0.111	5.188	0.452	3.502	2.268
(p)	(0.776)	(0.000)	(0.739)	(0.023)	(0.501)	(0.061)	(0.132)
Moran's I (Area)	-0.001	0.487	0.001	0.174	0.004	-0.217	-0.104
(p)	(0.239)	(0.000)	(0.226)	(0.000)	(0.082)	(0.114)	(0.085)
Obs.	50	50	50	50	21	11	50
OLS							
ln(Area)	-0.995	-0.995	-0.995	-0.995	-0.896	-0.983	-0.970
(Standard error)	(0.018)***	(0.018)***	(0.018)***	(0.018)***	(0.074)***	(0.064)***	(0.023)***
Constant	4.921	4.921	4.921	4.921	7.158	6.286	7.503
(Standard error)	(0.039)***	(0.039)***	(0.039)***	(0.039)***	(0.421)***	(0.310)***	(0.109)***
Adj. R^2	0.984	0.984	0.984	0.984	0.878	0.959	0.973

Case II shows the same rank-size distribution but ordered geographically along the coordinates. There is a significant spatial error influence confirmed by λ for the full distribution and a spillover effect ρ for the *Pareto* tail. The absolute value of α decreases substantially when incorporating spatial dependence. *Zipf's* law cannot be further confirmed for the upper (*Pareto*) tail, even though the random-generated distribution had been a *Pareto* (3,1) one. The strong positive spatial autocorrelation of the variable *Area* (*Moran's* I=0.487) substantiates this finding.

For case III spatial autoregressive effects (ρ) are only strong with respect to the full distribution of cities. This is to be explained by the spatial separation of big and small cities. Also here, spatial autocorrelation (*Moran's* I=0.429) of the variable *Area* is strong. In case of the big cities alone, there is no detectable spatial influence in the estimate. In both cases, the simple OLS regression as well as its extended spatial version, *Zipf's* law can be confirmed (α=0.995 for both versions).

For Case IV (geographically ranked bigger cities, normally distributed small cities) spatial error effects (λ) are highly significant. However, α remains rather stable independent of whether or not incorporating spatial effects. For the full distribution the the absolute value of α increases by 0.03, for the upper tail it slumps from 0.995 to 0.986.

The simulated distributions of coordinates show that, in theory, spatial distance may have a potential impact on the coefficient of the rank-size distribution of cities. The stylised models above are however rather artificial and not likely to be encountered anywhere in the real world. Therefore data of three EU countries are used to see how the spatial arrangement of cities may influence the estimate of rank-size distribution. As expected, the results generated are less spectacular than for the above cases II to IV but still suggesting spatial dependence to play a role in the rank-size distribution of cities for some countries.

In case of the Netherlands, estimated with 2011 radiance-calibrated DMSP-OLS night satellite images, a significant spatial influence can neither be established for the full distribution nor for the upper tail.

In case of Austria and Slovenia spatial effects can be detected for the upper tail (bigger cities). Interestingly, spatial autocorrelation of the variable *Area* is negative, hence big and small cities are directly related via spatial distance. This would suggest a polycentric urban structure. For Austria,

the spatial error coefficient (λ) is significant at the $p<0.05$ level. But the influence as such is negligible; the α coefficient remains stable and slumps just a little from 0.970 to 0.966. The Slovene case constitutes itself a bit different. Here spatial spillovers (ρ) are also significant at the $p<0.05$ level, the α coefficient, however, changes from 0.983 (*Zipf*) to 0.860. This might have been implied by the small number of observations being in line with *Zipf's* law for the upper tail. It is to be noted that especially for small samples (as for small countries) the simple OLS estimate of *Zipf's* law is affected by underestimated standard errors. For all three countries I additionally estimated *Zipf's* law of the upper tail by using the *Gabaix-Ibragimov* approach to more precisely sort out the deviation when introducing spatial dependence [23]:

$$\ln\left(R-\frac{1}{2}\right)=\ln(C)-\alpha\ln(S)+\varepsilon$$

The respective estimates are displayed by Table 3.

Table 3: Zipf's law for the Netherlands. Slovenia and Austria (upper tail) estimated by the Gabaix-Ibragimov OLS approach

	The Netherlands 2011 (DMSP)	Slovenia 2017 (VIIRS)	Austria 2017 (VIIRS)
Endogenous variable: ln(Rank-0.5)			
ln(Area)	-1.064	-1.178	-0.970
(Standard error)	(0.071)***	(0.074)***	(0.023)***
Constant	7.994	6.918	7.003
(Standard error)	(0.400)***	(0.349)***	(0.020)***
Adj. R^2	0.922	0.965	0.974

While in case of Austria there is no difference in the estimate, there are major differences when it comes to Slovenia and the Netherlands. Regarding the former case α jumps from 0.983 to 1.178, thus beyond confirmation of *Zipf's* law; in the latter case it changes from 0.896 (slightly below the margin of *Zipf's* law) to 1.064 (well in the acceptable range of *Zipf's* law). There is no noticable change in standard errors.

4. Conclusion and further interpretation

Compared to *Zipf's* law for words in languages the results suggest that in case of cities, their spatial arrangement matters: *Zipf's* law for cities behaves like *Zipf's* law for words only if small and big cities are normally distributed in space. In theory, during time cities may change their size, but the slope of the rank-size distribution remains rather stable [3, 24]. This is explained by its scale invariance and city growth independent of city size (*Gibrat's* law). Hence, the change of city ranks might be well explained by economic forces (structural change), but it is not directly visible in a changing slope of the rank-size distribution of cities. From a static point of view there is however reason to assume that specific location advantages and dispersion and concentration forces determine the spatial distribution of big and small cities. As soon as urban growth poles attract neighbour cities to grow, certain non-normally distributed patterns of spatial distribution of cities can evolve and spatial dependence of areal size increases (positive *Moran's I*). There is likewise the possibility of a more polycentric structure where big cities are functionally rather linked to smaller satellite towns. In this case, distance decay may outperform mass (size) and negative spatial dependence of areal size can be observed. If Gan et al. [2] were right, and *Zipf's* law represents nothing more than a pure statistical relationship, the extension of the model with spatial effects (i.e. different functional relations between big cities and the smaller urban centers of rural areas) would not change α. Where such spatial impact is significant, *Zipf's* law for cities is more than a pure statistical phenomenon.

References

1. Krugman P. Development, Geography and Economic Theory. MIT Press, Cambridge (Mass.). 1997

2. Gan L, Li D, Song S. Is the Zipf law spurious in explaining city-size distributions? Econ Lett. 2006; 92(2): p. 256-262

3. Gabaix X. Zipf Law for Cities: An Explanation.; Q J Econ. 1999; 114(3): p. 739-767

4. Fujita M, Krugman P, Venables AJ. The Spatial Economy: Cities Regions and International Trade. MIT Press, Cambridge (Mass.). 1999

5. Brakman S, Garretsen H, van Marrewijk C. The New Introduction to Geographical Economics. Cambridge University Press, Cambridge. 2009

6. Reggiani A, Nijkamp P. Did Zipf anticipate Socio-economic Spatial Networks? Environ Plann B. 2015; 42(3): p. 468-489

7. Zipf GK. Human Behaviour and the Principles of Least Effort. 1949; Addison Wesley, New York

8. Rosen KT, Resnick M. The Size and Distribution of Cities: An Examination of Pareto Law and Primacy. J Urban Econ. 1980; 8(2): p. 165-186

9. Brakman, S, Garretsen H, van Marrewijk C. The return of Zipf: Towards a further understanding of the rank-size distribution. J Reg Sci. 1999; 39(1): p. 183-213

10. Lalanne A. Zipf's Law and Canadian Urban Growth. Urb Stud. 2014; 51(8): p. 1725-1740

11. Rybski D, García Cantú Ros A, Kropp JP. Distance-weighted city growth. Phys Rev E. 2013; 87:04214

12. Le Gallo J, Chasco C. Spatial analysis of urban growth in Spain, 1900–2001. Empir Econ. 2008; 34: p. 59-80

13. Cheng K, Zhuang Y. Spatial Econometric Analysis of the Rank-size Rule for the Urban System: A Case of Prefectural-level Cities in China's Middle Area. Sci Geogr Sinica. 2012; 32(8): p. 905-912

14. Malevergne Y, Pisarenko V, Sornette D. Testing the Pareto against the lognormal distributions with the uniformly most powerful unbiased test applied to the distribution of cities. Phys Rev E. 2011; 83: 036111

15. Eeckhout J. Gibrat's Law for (All) Cities. Am Econ Rev. 2004; 94(5): p. 1429-1451

16. Alexander C. A City is not a Tree. Arch Forum. 1965; 122(1): p. 58-62

17. Jiang B, Miao Y. The Evolution of Natural Cities from the Perspective of Location-Based Social Media. Prof Geogr. 2014; 67(2): p. 295-306

18. Jiang B, Yin J, Liu Q. Zipf's Law for all the Natural Cities around the World. Int J Geogr Inf Sci. 2015; 29(3): p. 498-522

19. Bergs R. The detection of natural cities in the Netherlands – Nocturnal satellite inagery and Zipf's law. Rev Reg Res. 2018; 38(2): p. 111-140

20. Chen Y (2015) The Distance-Decay Function of Geographical Gravity Model: Power Law or Exponential Law? Chaos Soliton Fract. 2015; 77: p. 174-189

21. Bergs R, Issa M. What do night satellite images and small-scale grid data tell us about functional changes in the rural-urban environment and the economy? – Case studies Frankfurt-Rhein-Main and Ljubljana Urban Region, PRAC, Bad Soden. 2018. https://rural-urban.eu/publications/what-do-night-satellite-images-and-small-scale-grid-data-tell-us-about-functional

22. National Oceanic and Atmospheric Administration. Version 1 VIIRS Day/Night Band Nighttime Lights. 2017. https://ngdc.noaa.gov/eog/viirs/download_dnb_composites.html.

23. Gabaix X, Ibragimov R. Rank-1/2: A simple way to improve the OLS estimation of tail exponents. J Bus Econ Stat. 2011; 29(1): p. 24-39

24. Duranton G. Urban Evolutions: The Fast, the Slow, and the Still. Am Econ Rev. 2007; 97(1): p. 197-221
