# Supplementary material for: Spatial dependence in the rank-size distribution of cities – weak but not negligible
Source: PLoS One. 2021 Feb 9;16(2):e0246796. doi: 10.1371/journal.pone.0246796 (PMC7872244; doi:10.1371/journal.pone.0246796)
Supplement: Attachment — Submitted filename: Response to reviewers_16834.docx [file pone.0246796.s003.docx]

Re.: Spatial dependence in the rank-size distribution of cities

Rebuttal letter #20-16834

Dear editor and reviewers,

Thank you very much for your highly valuable critique and suggestions. In the revised manuscript I tried my best to address all your points raised. Originally, my paper had been a secondary statistical outcome of our ongoing research project on rural-urban interaction in the EU. I submitted the paper in May 2020 to the preprint server arXiv. Shortly afterwards I was kindly invited by PlosOne to submit my/our research to a forthcoming special issue on „Complex city systems“. In short, this is the background of my original paper. Inspired by your points raised I have now completely revised the manuscript, also using larger datasets for a better empirical foundation. I thus hope that the effort has helped to meet the expectations of the reviewers and the editor.

Reviewer 1:

1.There is really no economic model here; the model is purely statistical. The gravity model is not derived in this context from primitives, and is rather hokey. This is, in a way, a step backward.

*I fully agree with this view. It is not sufficient to determine a finding purely statistically when arguing from the viewpoint of economics. Zipf’s law is however extremely tricky when it comes to explaining it with economic theory (Krugman labeled the law as „spooky“). I now tried to solve the issue of the insufficiently explained relationship with gravity in a new section by first going back to Zipf’s own explanation of the „principle of least effort“ (lines 73-113) and then to link this very early insight (Zipf’s lemma) to the respective recent findings in the context of Central Places theory (with a reference to the suggested paper of Mori, Smith and Hsu and a second paper on that by Jiang). Both papers make clear that distance and spatial dependence matters in these Zipf-style regressions, so that they offer important justification to consider a spatial econometric approach (lines 119-138).*

2.The pre-Gabaix and post-Gabaix periods are distinguished by the presence of economic models in the post-Gabaix work. The problem with these models is often that they are designed to generate Zipf-like laws, and nothing else.

*This was a useful hint for me. My understanding is that Gabaix‘ model of normalized zero growth of cities determined by the underlying Gibrat’s law is only part of explaining scale invariance in Zipf’s law. The papers by Mori et al. (2020) and Jiang (2020) show that the functional differentiation of centrality matters, also with explanatory power for the fractal dimension of the law. But there seems even no contradiction between size independent (random) growth and a deterministic evolution of urban space. Therefore, for my argumentation I also added the important findings from Rybski‘s et al. paper on distance-weighted city growth that confirms scale invariance and Zipf’s law with „spatial explicit preferenial attachment“ (lines 139-159).*

3.This is not the first paper to notice the transition between Pareto at the top of the distribution and lognormal at the bottom (lines 55-69); Ioannides and Skouras, JUE, 2013. Not even cited.

*It would be a misunderstanding to interpret my formulations as a claim to be the first one noticing this peculiarity.This is not al all the case. For my earlier paper (Rev Reg Res 2018 38(2)) I had explored the well-known controversy between Malevergne et al. and Eeckhout. So, this knowledge obtained I just took over from that earlier research. Reading the paper by Ioannides and Skouras (2013) has much further enriched the argumentation to differentiate the distribution form of city rank-size.The JUE paper is now addressed and cited (lines 54-64).*

4. There is a theory for why distance should matter in these Zipf-style regressions, called Central Place theory, which is almost as famous as Zipf’s law. It is the long-time pursuit of Wen-Tai Hsu. See for a recent example, Mori, Smith and Hsu, PNAS, 2020, 117(12) 6469-6475.

*I fully agree. See my explanations on point 1 above*

5. Given the spatial dependence in rank R (the dependent variable) I wonder if there is also spatial dependence in population S, the independent variable.

*For the four country studies I now additionally tested spatial dependence of the predictor and the dependent variable in a Spatial Durbin model. This procedure did not properly work as estimates are mostly outside their allowed intervals. A simpler approach to address spatial dependence of the predictor is the Spatial-lag-of-X (SLX) model that directly shows spatial spillovers of the variable S by OLS estimation. In contrast to the SEM/SAR models, no significant spatial dependence was found for S. This finding is now documented by an additional table (lines 311-315) and two further paragraphs inserted (lines 211-217 and 346-348).*

6. The structure of spatial dependence in the regression should really be derived from the underlying economics

*This critique applies to every spatial econometric modelling that defines W simply as a contiguity or inverse distance matrix. Therefore, on the one hand, inverse distance that I used in my Stata exercises is admittedly not more than a proxy. A more realistic matrix reflecting the effort (real costs) to cover distance, given the heterogeneity of space, such as local topography or energy resources available, would be in fact a superior solution. However, the bottleneck for that is missing data. I have therefore added a clarifying paragraph stressing this general weakness (lines 218-227). On the other hand, a spatial econometric approach would shed light on the endogeneity of spatial distance in the Zipf regressions. This is a specific advantage, because here peculiar forms of deterministic spatial evolution (e.g. the German Ruhr area) suggest spatial dependence in the Zipf regressions already by viewing a map (clusters of big cities with often minor centrality relevance). In those cases, spatial dependence does not necessarily conceal a distribution that is not in line with Zipf (i.e. a false positive signal). On the contrary, as estimates for the US and Germany show, spatial dependence can also lead to estimates closer to Zipf’s law. Therefore, I additionally had a look at the city distribution in the upper tails (25 biggest cities for the USA, Germany and the UK, the 16 biggest for Slovenia) in terms of local spatial autocorrelation (lines 355-362). For the US and Germany there are many significant local Moran-I (LISA) coefficients, while this is not the case for the UK and Slovenia. Results of the local Moran I analysis thus correspond to the SEM/SAR estimates. A further figure on the LISA analysis was added to the paper (lines 353-354).*

Reviewer 2:

1. We are left wondering why focus on Netherlands, Slovenia and Austria rather than more widely studied countries like the US or even better a very comprehensive list of countries, ideally at several points in time. The more countries the better in my opinion, but if a selection is made the basis of that selection needs to be explained. Any statistical methodology is undermined if it is applied to an arbitrary subset of the potential data.

*I fully understand and agree to this concern. In fact, the research is funded by the European Commission in the context of a project dealing with rural-urban interaction covered by some EU country studies including Austria, Slovenia and the Netherlands where we had tested spatial size (segmented by cluster analysis of night satellite images) with Zipf’s law. I just used those data for the first manuscript. In addition to that, the EU funding of an open access paper should also be justified by the respective project context, e.g. the countries covered by that project. Hence, the selection had not been arbitrary. But still, the reviewer’s argument is admittedly too strong. Therefore, an alternative justification could be derived from the theoretical context elaborated, and hopefully this is convincing. I therefore explored databases on urban areas for three bigger countries (USA, UK and Germany) and left only Slovenia as a particular case of a young EU country (former province of Yugoslavia) for comparison purposes (Results for the Netherlands and Austria are now removed). It was, however, not possible for the three bigger countries to extract and transform the VIIRS night-light data in order to classify natural urban space. There are millions of observations in the highly resolved digital images for which outliers in such skewed pixel distributions need first to be removed by a quite demanding procedure. The computing capacity in our small institute is not sufficient for procedures with such extremely large datasets. Therefore, for the Zipf style regressions on the US, the UK and Germany I used population data as usual. Here I deemed it useful not to take cities proper but (functional) urban areas, because evidence has shown that urban areas much better represent a Zipfian distribution. A further comparative analysis over time was not possible given the limited time for the revision. But the original purpose of the paper was to show that there can be spatial dependence in Zipf’s law for cities rather than exploring change of spatial dependence over time in Zipf’s law. Nevertheless, such an extended comparative or panel approach combined with a more realistic spatial weight matrix (cf. Point 6 of reviewer 1) is now discussed as an interesting open question for future research on this thread of regional science (lines 224-227).*

2. I would like to see the results from simple rank-size regressions alongside the regressions that control for spatial dependence.

*These simple rank-size regressions had been (and still are) displayed in the tables alongside the spatial regressions including their interpretation. Those might have been either overlooked when reading the manuscript, or I have misunderstood the point raised. Nevertheless, I fully agree that without the simple rank-size regressions the respective spatial regressions would hardly be meaningful (see tables 1, 2 and 3: rows „OLS ln(Size)“ and the respective remarks in the section „Results“) .*

3. Would it be econometrically sensible to control for spatial dependence in Gabaix-Ibragimov regressions like those of table 3? If you cannot answer this question, it may be interesting to nevertheless run these regressions subject to appropriate disclaimers in order to allow direct comparisons of estimates.

*Yes, my earlier idea had been anyway to generally apply the Gabaix-Ibragimov approach for the country estimates, i.e. also for the ML estimator that is needed in the SEM/SAR regressions. This approach had been also pursued by le Gallo & Chasco (2008) in one of the very few spatial econometric studies on Zipf’s law. Estimates differ only minimally from the simple regression model. Now, all the country estimates displayed are Gabaix-Ibragimov ones (lines 271-272).*

4. I would prefer to see the discussion surrounding simulated data significantly condensed as I am not sure it adds much.

*I have deleted the simulations III and IV and just concentrated on the two potential extreme cases of normally distributed coordinates versus spatially ranked ones. This simulation should reveal the potential of spatial dependence in such Zipf regressions.*

5. It may be worth noting that combinations of lognormal-power laws similar to those studied in the simulations heve been studied by Ioannides & Skouras 2013

*As mentioned in the answer on point 3 of reviewer 1, the suggested JUE paper is now addressed and cited.*

6. No specific link to data is provided so I am not sure whether the source provided in the last sentence of section 2 is sufficient. I would prefer to see a link to the data actually used in the regressions (including to simulated data), not a link e.g. to a NOAA source from which the data were derived after extensive manipulation according to methods published elsewhere

*This is absolutely right. However, the guidelines say that „PLOS journals require authors to make all data necessary to replicate their study’s findings publicly available without restriction at the time of publication.“ During the online submission procedure I was therefore uncertain whether to attach the data files together with the manuscript or only once such a paper is accepted for publication. Shortly afterwards I contacted the editorial staff of PlosOne asking whether I can still upload the data for the peer review process. I was told that it will be fine to provide the full datasets once the manuscript has been accepted, so there was no need for me to do anything by then.*

*Reviewer 2 also remarked that estimates on Austrian, Slovene and Dutch natural urban space suggest minor to moderate spatial dependence. This is true, and I also believe that there is hardly any country where spatial dependence will change the estimate of the rank-size distribution to a large extent. The purpose of my paper was to show that there can be minor but significant spatial dependence; this is also confirmed by the estimates for the USA and Germany. It was less my intention to uncover major disturbance induced by spatial dependence. But with statistically significant spatial dependence Zipf’s law would not be a purely tautological phenomenon of a pre-determined spurious correlation as put forward by some authors.*
