# Supplementary material for: Spatial dependence in the rank-size distribution of cities – weak but not negligible
Source: PLoS One. 2021 Feb 9;16(2):e0246796. doi: 10.1371/journal.pone.0246796 (PMC7872244; doi:10.1371/journal.pone.0246796)
Supplement: Attachment — Submitted filename: Rebuttal2.docx [file pone.0246796.s005.docx]

Re.: Spatial dependence in the rank-size distribution of cities

Rebuttal letter #20-16834

Dear editor and reviewers,

thank you again for your further effort to review the revised version of my paper. The review reports are very encouraging. All suggestions are highly valuable and could be addressed in the revised manuscript. I hope this revised manuscript meets your expectations and look forward to your response. Below you find my answers separately on comments.

Reviewer #1 (including the suggestion of the editor):

" I think that the author has done as good a job as it is possible to do to address my comments regarding the empirical part of the paper. But I find the theory (Section 2) to be very annoying. It is not theory in a sense that a decent economist would recognize, as it is in the tradition of econophysics rather than mainstream economics. The econophysics models tend to be fairly mechanical models (including stochastic elements) rather than using an equilibrium based on individual optimization. A hint is that prices are nowhere to be found in this paper."

There is a well-developed theory with behavioral foundations, including notably Gabaix's QJE paper, which you cite, and material in Ch. 8 of Yannis M. Ioannides, From Neighborhoods to Nations, Princeton University Press, 2013. I think heeding Reviewer 2's critique will improve the paper enormously

Reply: I have now substantially revised and complemented section 2. Zipf’s law explained with early considerations by Zipf himself is now complemented by considerations of the economics of agglomeration. A useful starting point seemed to me the indivisibility of space (Starret’s spatial impossibility theorem) as a precondition for an equilibrium that cannot be explained by Zipf’s lemma alone. Here I found it worth to resort to the evolution of urban economic modeling from the 1970s to the new geographical economics since around 2000 to grasp the increasingly „spatial“ understanding of Zipf’s law from the economics viewpoint (in particular the more recent contributions of the Central Place theory). In addition to the paper of Gabaix (1999) I now discussed the relevant findings of Fujita et al (1999), Brakman et al. (2009) and those in more recent books and papers stressing the importance of Central Place theory for the explanation of Zipf’s law. Here I resorted to the book of the editor (Ioannides 2013), Hsu (2013), Mori et al. (2020) and Jiang (2017) [LINES 118-130; 142-182]. A useful argument for taking the path of spatial econometrics I also found in Dobkins and Ioannides (2001) on spatial interactions among US cities. [LINES 210-215].

Reviewer #2: This revision has addressed all the concerns I raised in my first report and the author has clearly made a serious effort at improving the quality of his paper.

However, the revision has also revealed some new problems which I summarize below:

1. My reading of the new empirical results is that the author is able to detect only a very weak impact of distance between cities on power exponent estimates in the four countries he examines. I think the author should say this more clearly (instead in the abstract, he states his finding as "distance matters"). The author should also avoid conflating distance with "spatial effects" - there may well be other spatial effects he has not tested for. There may also be distance effects in other countries or data, so the author should be more explicit that he is conducting an analysis with limited power and subject to significant type 2 error when interpreted as a test of "spatial dependence in the rank-size distribution of cities" (perhaps the title itself should be modified to reflect the more modest nature of the analysis). Summarizing, there is a little too much overselling for my taste, but this may be a style issue.

Reply: I agree with this comment. My original research interest was led by the idea that spatial dependence could potentially matter, and here the emphasis had been on the simulations which reveal major potential of such disturbances. Now the emphasis is on the country studies where spatial autocorrelation detected is weak but still partly significant. Hence I changed the title of the paper and the wording in a number of paragraphs, in particular to avoid suggesting something like globally valid relationships [Abstract; LINES 320-322; 444-449; 468-469]. I also revised the formulations conflating spatial effects with distance effects [various revisions of wording in the text; cf. track-change file].

2. The results of the second simulated data set in Table 2.II are puzzling. They suggest that even when the data really is generated to satisfy Zipf's law, the econometric approach used reveals a significant deviation from Zipf's law. This suggests a problem with the econometric method or its application or the data. Maybe I am missing something, but if so the author needs to explain.

Reply: With this simulation I deliberately aimed to generate a most powerful disturbance effect on the Zipf coefficient by maximising spatial autocorrelation. In the former manuscript this was only commented by a half-sentence. In the new version I added a further explanation that is in fact central in a way as it shows the theoretical potential of such effects [LINES 396-398]. By the way, with a repeated estimation to rule-out error I got the same results.

3. I am not fully comfortable with the author's description of the data used in the simulation. The author says he draws the "upper" 50 cities from a Pareto and the "lower" 59 from a lognormal. If the draws are really random, the largest lognormal draw could be larger than the smallest Pareto draw, but the phrasing suggests this cannot happen, or at least did not happen in the two draws the author used. The author should explain this more clearly and make sure he isn't choosing a sample with the properties he wants. While I don't expect the author to do this at this stage, the proper way to simulate this data would be from a single distribution which had both a lognormal and a pareto component.

Reply: I have now explained how this hybrid distribution was generated. My original idea had been to generate just one typical rank-size distribution of cities and, as long as this can be sufficiently rigged by distorting the location of cities via the coordinates, the exercise would prove the potential existence of spatial dependence in Zipf’s law. It is right that the lower (lognormal) tail could be also much larger. This would have an influence on the estimation of the entire distribution, however not when looking at the upper Pareto tail. This is now explained in section 4 [LINES 289-296].

4. It should be made clearer that the value of the simulations is to demonstrate that the chosen econometric methodology is powerful enough to detect distance effects if the patterns are sufficiently strong. In my view the simulations are purely a prelude to motivate the empirical analysis.

Reply: I fully agree with that. The issue is now clarified [LINES 68-70].

5. I would like to see more detailed table legends, so that tables can be interpreted without having to refer to the text. We have to guess what Columns I and II mean. Please explain each item in the table in detail in the legend - a little spoon feeding for the reader can only help.

Reply: I agree to that and added respective explanatory notes to Tables 1 and 2 [LINES 354-357; 368-371].

Further corrections

In addition to revisions suggested by the reviewers and the editor I also corrected the SEM and SAR estimates of the four countries and the respective LISA figures. For the population based estimations it was necessary to change the geographical projection of the raw data into an equal area one. The correction reveals only negligible differences, most of them even improving the estimates expected [LINES 374-375; 431-432]. In addition some few transcription errors were corrected. There was no need to change the projection for the simulations and the estimation on segmented natural cities in Slovenia. In those cases, coordinates already represent an equal area projection. There was also no need to modify the projection of the SLX estimates as the respective Stata command automatically calculates the true distances by the Vincenty formula. For Slovene municipalities (>10,000 inhabitants) the SLX estimation was to be corrected because of a wrong entry for the distance threshold. But this has not changed the insignificant result.

Some few further style and orthographic corrections can be identified by inspecting the track-change version of the manuscript.
